# HPV Vaccination in Kenya: The Challenges Faced and Strategies to Increase Uptake

**DOI:** 10.3389/fpubh.2022.802947

**Published:** 2022-03-21

**Authors:** Christine Muthoni Karanja-Chege

**Affiliations:** Kenyatta University, Nairobi, Kenya

**Keywords:** Human Papilloma Virus (HPV), girls, Kenya, vaccine, uptake, adolescent

## Abstract

Human papilloma virus (HPV) is the leading cause of ano-genital cancers globally with cervical cancer as the top cause of cancer- related deaths in women. Over 90% of these deaths occur in low income countries where cancer control strategies remain inadequate. HPV vaccination provides protection against HPV types 16 and 18 which are responsible for approximately 70% of cervical cancer cases. The optimal age of vaccination is in the early adolescent period, before sexual debut with possible HPV infection. Studies have shown that children residing in low income settlements are at risk of early initiation of sexual activity. Adolescent vaccination programs would provide an avenue to link other health promotion strategies targeting this age group that has hitherto been left out of many health interventions in 2019, Kenya introduced HPV vaccine to be given to 10 year old girls. Uptake has been sub-optimal with only 33% of targeted population receiving the first dose in 2020 and 16% returning for the 2nd dose. While disruption of immunization programs by the COVID-19 pandemic contributed to the low coverage, other factors such as low demand fuelled by misinformation have also played a role.

## Importance of This Review Paper

Vaccination is one of the most important and equitable public health strategies in existence to combat infectious diseases globally (1). Persistent Human Papilloma Virus (HPV) infection in the genital tract is the leading cause of anogenital carcinomas (2). Cervical cancer is of special concern because of the internal location of the cervix as well as the occult cancer presentation in early stages, implying that active screening in the at-risk women must be done regularly to detect pre-cancerous and early stage cancer lesions. In most low-income countries, access to centers that carry out screening and diagnosis is limited and awareness of the need for regular pap-smears is also low leading to many women presenting with advanced cervical carcinoma.

HPV vaccines have been shown to be effective in preventing HPV infection when administered in young boys and girls before their sexual debut. In countries with high vaccine coverage such as Australia which reported a HPV vaccine coverage of 80% in females and 76% in males in 2019 (3), a significant decline in vaccine type-HPV infection and high grade precancerous cervical lesions has been reported with a substantial impact on cervical cancer incidence expected in the coming years (4). Routine HPV vaccination was introduced in Kenya in late 2019 to 10 year old girls. Uptake has been sub-optimal owing to various challenges that we seek to elucidate in this review.

## Background of HPV Associated Illnesses, HPV Vaccines and Adolescent Vaccination

Human Papilloma Viruses comprise over 100 serotypes of which about 14 are of oncogenic potential. These high-risk viruses are sexually transmitted and cause a majority of oral and ano-genital cancers in both males and females. The non-oncogenic types cause cutaneous and mucosal warts including sexually transmitted oropharyngeal and ano-genital warts (5). Cervical carcinoma, one of the HPV-related cancers is of great public health concern as it is the leading cause of cancer deaths in females world-wide. In 2020, there were over half a million cases of cervical cancer globally with over 300,000 deaths. Ninety percent of these deaths occur in low income countries where cancer control strategies remain inadequate (6). In 2020, Kenya reported over 5,000 new cases of cervical cancer and about 3,000 deaths from cervical cancer complications (7).

HPV vaccination is one of the primary prevention approaches in the comprehensive cancer control strategy used in tandem with promotion of abstinence, faithfulness to one partner and condom use. Adolescent vaccination is one of the strategies toward implementing the life-course immunization approach set out in the Immunization Agenda 2030. This approach expands the coverage of routine immunization to other age groups outside infancy and also seeks to improve coverage in under-served populations under the mantra “reaching everyone, everywhere” (8).

Young adolescents, classified as children between 10 and 15 years, have been left out of many public health programs and yet remain vulnerable to many health threats. Promotive and preventive interventions targeted toward this group are likely to have a greater impact on future health and well-being with far-reaching implications on productivity across generations.

Recent studies have shown that the age of sexual debut is earlier than that depicted in past reports drawn from census data that classifies adolescents in the 15–24 years age bracket, missing the younger adolescents. A study carried out in 2013 among boys and girls aged 12–16 years, living in two large informal settlements in Nairobi Kenya, further explored the predictors of early sexual debut. It emerged that family dysfunction and lack of parental supervision were leading factors associated with early engagement in sexual intercourse. Adolescents enrolled in schools were found to be protected from early sexual debut (9).

Many of the young adolescent girls in hard-to-reach populations such as low income settlements and nomadic communities do not attend school regularly and reaching them with HPV vaccination was anticipated to present a substantial challenge. A purely school based approach to vaccination would fail to capture them and hence the government decided to use a facility based approach with community and school mobilization by teachers and other identified community members (10). Stakeholders involved in the roll out had to come up with strategies to address potential barriers. The key strategies employed to sensitize the community included getting community leaders to endorse the vaccine in collaboration with the government and providing simple but accurate information to the community using creative and engaging methods such as skits and media talk shows.

Promoting behavior change as a lone strategy for preventing sexually transmitted infections has been shown to be ineffective in reducing transmission and would hence not be effective for prevention of HPV infection as well. Behavior change must be linked to other prevention strategies such as condom use, vaccination and screening (11).

There are 3 vaccines currently approved for prevention of HPV. All the three types offer protection against HPV serotypes 16 and 18 that cause 70% of cervical cancer with the latest nanovalent HPV vaccine offering an additional 20% protection against 31, 33, 45, 52 and 58 which are other high risk serotypes for ano-genital cancers. The quadrivalent and nanovalent HPV vaccines also protect against types 6 and 11, responsible for ano-genital warts as well as the rare vertically transmitted respiratory papillomatosis.

HPV vaccines are licensed for use in both boys and girls between 9 and 26 years of age, with the optimal age of administration being 9–14 years of age. This is considered to be the most appropriate age for vaccination as most of the children have not had any sexual encounters by then and therefore unlikely to have acquired HPV infection (12).

## HPV Vaccine Pilot in Kenya

HPV vaccines have been in use in the private sector in Kenya since 2006. In 2013–2015, a pilot vaccination program was conducted by the Ministry of Health in Kitui county, eastern Kenya. Over the two-year period, 22,500 girls in class 4 who were aged between 9 and 12 years received 2 doses of the HPV vaccine. The grades-based approach was found to be more suitable so as to capture a large cohort of eligible children in one class. This successful uptake of 96% was used as an indicator of the country's readiness to roll out the HPV vaccine and incorporate it into the routine immunization schedule, beginning with 10 year old girls with a plan to increase the scope of coverage sequentially when more doses became available (13). The pilot vaccination program in Kitui county was school-based and this is likely the main reason for the high coverage reported. Rwanda, another eastern African country reported a similarly high uptake of 95% in 2011 when they introduced HPV vaccination for 10–14 year old girls using a school-based program (14).

A study evaluating the acceptabilty of a school based vaccination program among teachers in Kitui was conducted in 2015 during the HPV pilot vaccination program. The major findings were that most teachers (89%) were supportive of the school based approach to administering the HPV vaccine. High acceptability was correlated with high levels of awareness regarding the availabilty of the vaccine (15). It is therefore imperative that teachers be supported to deliver accurate information regarding the HPV vaccine to bolster confidence in recipients and their care-givers.

Schools are considered better for vaccination programs targeting school-age children and adolescents. There is increased acceptability among parents and caregivers as teachers are seen as trusted custodians of children and schools as safe havens for the welfare of children. Teachers' knowledge and attitude about vaccination therefore plays a major role in ensuring success of school based vaccination programs.

## HPV Vaccine Introduction in Routine Immunization and the Challenges Faced

In late 2019, Kenya joined 115 other countries that had already commenced vaccination. HPV vaccination was to be implemented using a blend between facility, community and school based strategies. Communication and social mobilization was to be primarily carried out in schools and the community, while vaccine administration was to be done in the health facilities. This approach was favored over the purely school-based system that had been employed in the 2013 Kitui pilot for two major reasons. One of the reasons was to capture the eligible girls who were not enrolled in school and the other was that a school based vaccination programme was likely to be encumbered by logistical difficulties and high cost (10). Given that the HPV vaccine was to be given to a new target population that had not been previously included in the routine immunization schedule, a broad array of stakeholders had to undergo capacity building to ensure successful introduction of the new vaccine. These stakeholders were drawn from the ministry of education staff including teachers, community health volunteers, adolescent health professionals, among others. Community health volunteers were considered essential in reaching girls not enrolled in schools especially in marginalized communities. Advocacy, social mobilization and communication activities were conducted using diverse social and mainstream media platforms such as radio and television shows. Information, Education and Communication (IEC) materials were developed in both English and Swahili targeting key audiences with simple messages regarding the HPV vaccine (16).

One of the first challenges that the program encountered was calculating and establishing the target population. Young adolescents typically have no designated routine clinics and screening programs and so are not captured in health systems data. The ministry of education data was also found to be inaccurate and hence not useful in determining the vaccination cohort numbers. The National Vaccination and Immunization Program (NVIP) therefore had to turn to partners such as John Snow, Inc. (JSI), a public health research and consulting organization, to help with microplanning and mapping strategies at county and sub-county levels. This meant getting the accurate data directly from schools within a health facility catchment area and working with school teachers to sensitize parents to take their eligible daughters for vaccination at the nearest health facility (16).

Despite the robust effort to prepare and inform the public about the safety, efficacy and benefit of having young girls receive the HPV vaccine, and a colorful flagship ceremony graced by the President of Kenya and other dignitaries, dissenting opinions began to emerge. The loudest and most influential vaccine opponents came from the Catholic Church which is an important societal voice as one fifth of Kenyans profess to belong to the Catholic faith according to a survey done. A section of doctors allied to the Kenya Catholic Doctors' Association took to the media urging parents not to allow their 10 year old girls to receive the vaccine. Among their myriad claims were doubts about safety and efficacy, citing evidence unbacked by science from certain internet channels linked to anti-vaccination groups. They cautioned Kenyans that allowing the youth to receive vaccination against HPV which is sexually transmitted was tantamount to giving them free reign to engage in pre-marital sex which is proscribed by the Christian doctrine. They urged the medical fraternity to instead focus on promoting abstinence among the unmarried, faithfulness to one sexual partner within the confines of marriage and cervical cancer screening tests for sexually active women (17).

A qualitative study carried out among main stakeholders out in two counties in the North Eastern and coastal regions of Kenya sought to establish their knowledge, attitudes and practice regarding HPV infection and vaccination. Findings revealed that knowledge gaps among the interviewees were the largest contributor to vaccination refusal (18). It is therefore imperative that all stakeholders should be equipped with accurate information regarding HPV infection as a cause of cervical cancer as well as how vaccination provides protection.

The COVID 19 pandemic that struck in the early months of 2020 necessitated prolonged school closure and also caused significant disruption to health service delivery. Immunization services were especially hard hit as priority shifts necessitated by the COVID-19 pandemic response resulted in a number of counties postponing some scheduled immunization activities such as polio and Measles/rubella campaigns (19). Faced with the dire threat of reversal of the gains made in raising HPV vaccine coverage, the ministry of health through the National Vaccines and Immunization Program embarked on conducting HPV vaccine catch-up through outreach programs which were conducted in open spaces in-line with COVID 19 infection control protocols.

## HPV Vaccine Uptake and Response to the Challenges

Following the negative sentiments expressed by various prominent opinion leaders, the public became increasingly divided and confused as to the safety of the HPV vaccine for their 10 year old girls. Immunization stakeholders had to act fast to counter the negative narrative that threatened to disrupt the national roll- out of the HPV vaccine in 2019.

The National Vaccines and Immunization program (NVIP) in collaboration with the Kenya National Immunization Technical Advisory Group (KENITAG) and a consortium of Civil Societies under the umbrella organization Immunization Advocacy Initiative (IAI), urgently convened to come up with multi-stakeholder advocacy strategies aimed at countering misinformation regarding HPV vaccine and disseminating accurate scientific facts regarding safety and efficacy of the vaccine (20). To bolster the public's confidence, real world stories from other countries such as Australia that had introduced HPV vaccines into their routine immunization schedules and eliminated cervical cancer were shared (21).

Technical experts on immunization from various professional bodies including the Kenya Pediatric Association (KPA) participated in the media engagements that were broadcasted through national as well as local media stations targeting Kenyans both in the rural and urban areas with information tailored to varying literacy levels (22). Face to face consultative meetings between the technical experts, policy makers as well as members of the clergy were held leading to the eventual public endorsement of the vaccine as safe and necessary by this influential group of public leaders (23).

This coordinated campaign effort showed initial success. In parts of Western Kenya, health workers reported that girls were actually demanding HPV vaccination even in situations where their parents expressed hesitancy. Unfortunately, in other parts of the country especially those close to the capital with higher rates of social media use showed a dismal response. The aspersions cast on the safety of HPV vaccines by societal leaders were shared widely on social media and amplified many times over by vocal pseudoscience protagonists expressing anti-vaccine sentiments and conspiracy theories (24).

A significant proportion of eligible girls missed vaccination with many parents adopting a wait and see approach while others refused vaccination altogether.

Vaccine hesitancy was also witnessed among the health care workers, many of whom belong to various religious faiths that were responsible for propagating negative sentiment regarding the HPV vaccine. Their lack of confidence manifested in a reluctance to actively promote the vaccine to eligible girls, failure to provide enquiring parents with correct information on the risks and benefits associated with HPV vaccination; choosing to vaccinate girls whose parents expressed minimal hesitancy and therefore consented fully to vaccination.

Notwithstanding these challenges, HPV vaccine uptake has improved over time as focused advocacy initiatives continue in the 47 counties of Kenya harnessing the strength of community health workers (25).

Uptake of the first dose of HPV vaccine improved from 25% in 2019 to 33% in 2020. In 2020, it was however reported that only just over 110,000 girls returned for the 2^nd^ dose, representing 16% coverage. The reasons for drop out need to elucidated further but may include forgetting due to the long 6 month dose interval, assumption that one dose is sufficient as well as disruption of Immunization services by the COVID-19 pandemic (26).

In November 2021, the Ministry of Health through NVIP commenced Periodic Intensification of Routine Immunization (PIRI) activities to improve coverage that had been significantly affected by the COVID-19 pandemic. This campaign is to run for 100 days till early February, in all the country's 47 counties. There is an ambitious target of improving the HPV coverage of girls aged 10–14 years to 70%. In order to reach the target population, mapping of schools and linking them to health facilities has been done. Trained teams carrying out vaccination in schools report that uptake is good and many parents have signed consent forms for their eligible girls to receive vaccination (27, 28).

## Lessons for the Future

HPV vaccination offers a golden opportunity to link young adolescents into promotive and preventive health services. Young adolescents (10–15 years) have been consistently left out of many health initiatives mainly due to the fact that they are an age-group that exhibits relatively few vulnerabilities to health compared to under-fives and older adolescents (29). Linking sexual health education to HPV vaccination has the possible benefit of delaying sexual debut as well as equipping the young adolescents with skills to navigate the difficult teenage years by making the right choices. This would in turn increase their chances of staying in school with better prospects for future livelihood.

Vaccine hesitancy is a growing threat to immunization programs world-wide. In Kenya, as in many other countries, the reasons for the waning vaccine confidence are highly context specific and should therefore not be addressed in a uniform approach. HPV vaccine introduction in Kenya continues to be a learning point for all immunization stakeholders on how to ensure successful uptake of new vaccines, and especially where a new cohort is involved. Despite having done a lot of prior social mobilization and advocacy, confidence in the HPV vaccine was severely compromised by rumors and misconception. It became evident that continuous engagement and public multi-stakeholder dialogue should be employed at all stages of vaccine introduction.

Unforeseen shocks such as the COVID 19 pandemic can reverse gains in immunization coverage and even threaten the successful introduction of new vaccines. Leaders in health and policy makers must work together to build resilience in Immunization programs to enable them to withstand disruption.

## Author Contributions

The author confirms being the sole contributor of this work and has approved it for publication.

## Conflict of Interest

The author declares that the research was conducted in the absence of any commercial or financial relationships that could be construed as a potential conflict of interest.

## Publisher's Note

All claims expressed in this article are solely those of the authors and do not necessarily represent those of their affiliated organizations, or those of the publisher, the editors and the reviewers. Any product that may be evaluated in this article, or claim that may be made by its manufacturer, is not guaranteed or endorsed by the publisher.
